# Optimal Control of the Lost to Follow Up in a Tuberculosis Model

**DOI:** 10.1155/2011/398476

**Published:** 2011-10-11

**Authors:** Yves Emvudu, Ramsès Demasse, Dany Djeudeu

**Affiliations:** Laboratory of Applied Mathematics, University of Yaoundé 1, P.O. Box 812, Yaounde, Cameroon

## Abstract

This paper deals with the problem of optimal control for the transmission dynamics of tuberculosis (TB). A TB model that considers the existence of a new class (mainly in the African context) is considered: the lost to follow up individuals. Based on the model formulated and studied in the work of Plaire Tchinda Mouofo, (2009), the TB control is formulated and solved as an optimal control theory problem using the Pontryagin's maximum principle (Pontryagin et al., 1992). This control strategy indicates how the control of the lost to follow up class can considerably influence the basic reproduction ratio so as to reduce the number of lost to follow up. Numerical results show the performance of the optimization strategy.

## 1. Introduction

Cameroon has a high rate of tuberculosis endemic. It is estimated that in absence of effective epidemiology statistics, there are 100 new Cases for 100 000 habitants per year [1]. Like in many sub-Saharian African countries, the fight against tuberculosis (TB) in Cameroon is difficult due to the interaction with the Human Immunodeficiency Virus (HIV) [2] and particularly with the poor social-economic conditions.

In the literature, there are many TB mathematic models [3–5]. The study of those models has an impact in the control process of the disease. Most of those models are SEIR-models; for those models, one supposes that the population is subdivided in four epidemiological classes: Susceptible individuals, latently infected individuals (those who are infected but not yet infectious), infectious, and the recovered or cured individuals. The particularity of those type of models is that, the rate at which susceptible individuals become latently infected or infectious is a function of infectious individuals number in a population at that time.

In this paper, we study a TB model adapted in the African context in general, particularly for Cameroon. In this model, we take in account the susceptible low and fast progression to latently infected and infectious classes, respectively. We also take into account infectious individuals on chemoprophylaxis, and we introduce a constant rate to become a cured individual.

We note that, the statistic studies [6] prove that many infectious patients do not take their treatment until the end due to a brief relief or a long time for complete treatment. Otherwise, some of those individuals can transmit the disease without presenting any symptom. In this work, we call them lost to follow up individuals (those who have active TB but are not in a care center) [7]. In Cameroon, for example, for a national program of fight against TB, there is about 10% of infectious individuals who do not end their treatment and become lost to follow up individuals. The class of lost to follow up individuals has already been considered by some authors [5, 7]. Previous [5, 7] works clearly show that the progression toward the lost to follow up class has a negative effect on the host population. For that, lost to follow up individuals are very dangerous for human health because they are able to transmit the disease very quickly and discreetly. For our knowledge, the previous authors did not address the question of controlling the evolution to the lost to follow up class. In this paper, we address the question to do so. Our control policy based on decreasing the number of people going to the class of lost to follow up individuals. We first formulate a mathematical model taking into account our control functions. Then, we perfect a mathematical analysis of the model where we compute the basic reproduction ratio of the controlled system. We then define a cost function so that we could deduce the optimal control function. A huge part of this work is to compute the solutions numerically and then draw a conclusion about the efficiency of the control.

## 2. The Model

We present a tuberculosis model that incorporates the essential biological and epidemiological features of the disease such as exogenous reinfection and chemoprophylaxis of latently infected individuals.

We consider a population of *N* people. We assume that latently infected individuals (inactive TB) have a variable (typically long) latency period. At any given time, an individual is in one of the following four states: susceptible, latently infected (i.e., exposed to TB but are not infectious), infectious (i.e., has active TB but is in a care center), and lost to follow up (i.e., has active TB but is not in a care center). We will denote these states by *S*, *E*, *I*, and *L*, respectively. Any recruitment is into the susceptible class and occurs at a constant rate Λ. The transmission of tuberculosis occurs following an adequate contact between a susceptible and infectious or lost to follow up. We assume that a fraction *δ* of the lost to follow up are still infectious and can transmit the disease to susceptible individuals (some of them could die or recover). On an adequate contact with infectious or lost to follow up, a susceptible individual becomes infected but not yet infectious. This individual remains in the latently infected class for some latent period. We use the standard mass balance incidence expressions *βSI* and *βδSL* to indicate successful transmission of TB due to nonlinear contact dynamics in the population by infectious and lost to follow up, respectively. The fractions *p*_1_ and *p*_3_ of the newly infected individuals are assumed to undergo fast progression directly to the infectious and lost to follow up classes, respectively. The remainders *p*_2_ = 1 − *p*_1_ − *p*_3_ are latently infected and enter the latent class. After receiving an effective therapy, individuals leave the infectious class *I* to the latently infected class *E* at a rate *r*_2_. We assume that chemoprophylaxis of latently infected individuals reduces their reactivation at a constant rate *r*_2_. We also assume that individuals leave the lost to follow up class *L* to the latently infected class *E* with a constant rate *γ*_2_. This can be due to the response of the immune system or traditional treatment (via a traditional practitioner). Another assumption is that among the fraction 1 − *r*_2_ of infectious who did not recover, some of them who had begun their treatment would not return to the hospital for the examination of sputum at a constant rate *ϕ* and enter the class of lost to follow up *L*. After some times, some of them will continue to suffer from the disease and will return to the hospital at a constant rate *γ*_1_. We assume that the chemoprophylaxis of latently infected individuals *E* reduces their reactivation at rate *r*_1_. Thus, a fraction (1 − *r*_1_)*E* of infected individuals who do not receive effective chemoprophylaxis become infectious and lost to follow up with a constant rate *K*_1_ and *k*_2_, respectively (low progression of the disease). The constant rate for non-disease-related death is *μ*, thus 1/*μ* is the average lifetime. Infectious and lost to follow up have additional death rates due to TB-induced mortality with constant rates *d*_1_ and *d*_2_, respectively.

**Figure 1:**
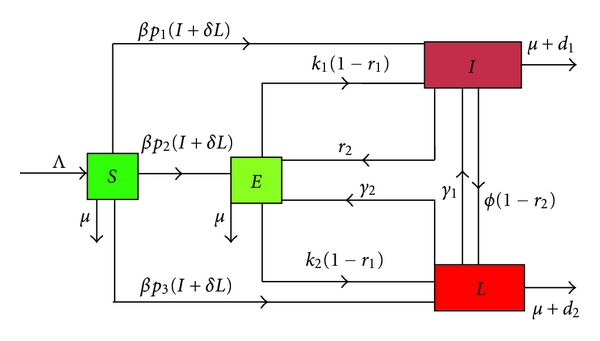
Flow diagram of the model without control.

 Thus, the corresponding transfer diagram is [7] illustrated in Figure 1.

We have *N* = *S* + *E* + *I* + *L* individuals. And the not listed parameter in the previous paragraph is as follows.

***β*: Transmission Rate**

The above scheme leads to the following differential system:

(1) $$\begin{array}{c} \dot{S}=\Lambda -\mu S-\beta \left(I+\delta L\right)S,\\ \dot{E}=\beta p_{2}\left(I+\delta L\right)S+\gamma_{2}L+r_{2}I-\left[\mu +\left(k_{1}+k_{2}\right)\left(1-r_{1}\right)\right]E,\\ \begin{array}{cc} \dot{I} & =\beta p_{1}\left(I+\delta L\right)S+k_{1}\left(1-r_{1}\right)E+\gamma_{1}L\\ & \;-\left[r_{2}+\mu +d_{1}+\Phi \left(1-r_{2}\right)\right]I, \end{array}\\ \begin{array}{cc} \dot{L} & =\beta p_{3}\left(I+\delta L\right)S+k_{2}\left(1-r_{1}\right)E+\Phi \left(1-r_{2}\right)I\\ & \;-\left(\gamma_{1}+\gamma_{2}+\mu +d_{2}\right)L. \end{array} \end{array}$$

### 2.1. The Control and Its Policy

The aim of the control is to decrease the total number of the lost sight patients during a period of time *t*_*f*_. The strategy of control we adopt consists of introducing two control parameters *u*_1_(*t*) and *u*_2_(*t*) representing the following.

*u*_1_: The effort made to take systematically the infectious patients in a health center in charge.
*u*_2_: The effort made to take systematically the latently infected people declared infectious in charge.

Having introduced the functions *u*_*i*_(*t*); *i* = 1,2, we obtain the following compartmental model.

**Figure 2:**
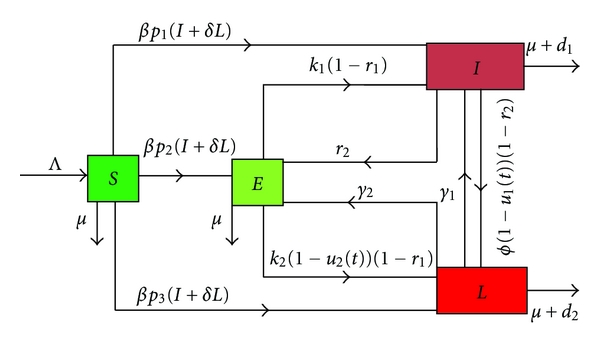
Flow diagram of the model with control.

Figure 2 leads us to the following differential system:

(2) $$\begin{array}{c} \dot{S}=\Lambda -\mu S-\beta \left(I+\delta L\right)S,\\ \begin{array}{cc} \dot{E} & =\beta p_{2}\left(I+\delta L\right)S+\gamma_{2}L+r_{2}I\\ & \;-\left[\mu +\left(k_{1}+k_{2}\left(1-u_{2}\left(t\right)\right)\right)\left(1-r_{1}\right)\right]E, \end{array}\\ \begin{array}{cc} \dot{I} & =\beta p_{1}\left(I+\delta L\right)S+k_{1}\left(1-r_{1}\right)+\gamma_{1}L\\ & \;-\left[r_{2}+\mu +d_{1}+\Phi \left(1-u_{1}\left(t\right)\right)\left(1-r_{2}\right)\right]I, \end{array}\\ \begin{array}{cc} \dot{L} & =\beta p_{3}\left(I+\delta L\right)S+k_{2}\left(1-u_{2}\left(t\right)\right)\left(1-r_{1}\right)E\\ & \;+\Phi \left(1-u_{1}\left(t\right)\right)\left(1-r_{2}\right)I-\left(\gamma_{1}+\gamma_{2}+\mu +d_{2}\right)L. \end{array} \end{array}$$

With initial conditions (*S*(0); *E*(0); *I*(0); *L*(0)) ∈ ℝ_+_^4^.

We set

(3) $$\begin{array}{cc} & \left[\dot{S};\dot{E};\dot{I};\dot{L}\right]\\ & :=\left[g_{1}\left(S,E,I,L\right);\;g_{2}\left(S,E,I,L\right);\;g_{3}\left(S,E,I,L\right);\;g_{4}\left(S,E,I,L\right)\right], \end{array}$$

where the functions *g*_1_, *g*_2_, *g*_3_, and *g*_4_ are defined by the right-hand side of the system (2).

**Remark 1**

The functions *u*_*i*_(*t*); *i* = 1, 2 are assumed to be integrable in the sense of Lebesgue, bounded with (0 ≤ *u*_*i*_(*t*) ≤ 1). When the functions of control are near to 1, the control is very strict.

## 3. Mathematical Analysis of the Model with Control

System (2) can be written in the following compact form:

(4) $$\begin{array}{c} \begin{array}{c} \dot{S}=\varphi \left(S\right)-S\langle \eta ,Y\rangle ,\\ \dot{Y}=S\langle \eta ,Y\rangle B+A\left(t\right)Y, \end{array} \end{array}$$

where *S* is a state representing the compartment of susceptible individuals and *Y* = (*E*,*I*,*L*)^*T*^ is the vector representing the state compartment of different infected individuals (latently infected individuals, infectious, lost to follow up individuals). *φ*(*S*) = Λ − *μS* is a function that depends on *S* ∈ ℝ_+_, *η* = (0,*β*,*βδ*)^*T*^, *B* = (*p*_2_, *p*_1_, 1 − *p*_1_ − *p*_2_), 〈, 〉 is the usual scalar product in ℝ^3^, and *A* is a Metzler [8] 3 × 3 nonconstant matrix defined as

(5) $$\begin{array}{c} A\left(t\right)=\left[\begin{array}{ccc} -a_{11}\left(t\right) & r_{2} & \gamma_{2}\\ k_{1}\left(1-r_{1}\right) & -a_{22}\left(t\right) & \gamma_{1}\\ a_{31}\left(t\right) & a_{32}\left(t\right) & -a_{33}\left(t\right) \end{array}\right] \end{array}$$

with

(6) $$\begin{array}{cc} & a_{11}\left(t\right)=\mu +\left(k_{1}+k_{2}-k_{2}u_{2}\left(t\right)\right)\left(1-r_{1}\right),\\ & a_{22}\left(t\right)=r_{2}+\mu +d_{1}+\phi \left(1-u_{1}\left(t\right)\right)\left(1-r_{2}\right),\\ & a_{31}\left(t\right)=\left(k_{2}-k_{2}u_{2}\left(t\right)\right)\left(1-r_{1}\right),\\ & a_{32}\left(t\right)=\phi \left(1-u_{1}\left(t\right)\right)\left(1-r_{2}\right),\\ & a_{33}\left(t\right)=\gamma_{1}+\gamma_{2}+\mu +d_{2}. \end{array}$$

**Remark 2**

The dynamic of the susceptibles is asymptotically stable. In other words, for the system

(7) $$\begin{array}{c} \dot{S}=\varphi \left(S\right) \end{array},$$

there exists a unique equilibrium *S*_0_ = Λ/*μ* such that

(8) $$\begin{array}{c} \varphi \left(S\right)>0\;\text{for }0<S<S_{0},\\ \varphi \left(S\right)<0\;\text{for }S_{0}<S. \end{array}$$

### 3.1. Positive Invariance of the Nonnegative Orthant

We have the following result.

**Proposition 3**

The nonnegative orthant ℝ_+_^4^ is positively invariant for the system (4).

**Proof**

The system (4) can be written as

(9) $$\begin{array}{c} \dot{S}=\varphi \left(S\right)-S\langle \eta ,Y\rangle ,\\ \dot{Y}=\left(SB\eta^{T}+A\left(t\right)\right)Y. \end{array}$$

The fist equation of system (9) implies that

(10) $$KS\left(t\right)=KS_{0}e^{-K(t-t_{0})}+\Lambda \left(1-e^{-K(t-t_{0})}\right)$$

for *t* ≥ *t*_0_.

With *K* = *μ* + *β*(*I* + *δL*). For *I* ≥ 0, *L* ≥ 0, and *S*_0_ ≥ 0, it comes that *S*(*t*) ≥ 0 for all *t* ≥ *t*_0_. As a consequence, ℝ_+_ is invariant for the system $\dot{S}=\varphi \left(S\right)-S\langle \eta ,Y\rangle$. For *S* ≥ 0, the matrix (*SBη* ^*T*^ + *A*(*t*)) is a Metzler matrix. Since it is well known that linear Metzler matrices let invariant the nonnegative orthant, this proves the positive invariance of the nonnegative orthant ℝ_+_^4^ for the system (4).

### 3.2. Boundedness of Trajectories

Adding all equations of model (2), one has

(11) $$\dot{N}\left(t\right)=\Lambda -\mu \left(S+E+I+L\right)-d_{1}I-d_{2}L.$$

Thus, one can deduce that

(12) $$\dot{N}\left(t\right)\leq \Lambda -\mu N\left(t\right).$$

After integration, using the constant variation formula, we have

(13) $$N\left(t\right)\leq \frac{\Lambda }{\mu }+e^{-\mu t}N\left(0\right).$$

It then follows that

(14) $$\underset{t\to +\infty }{\lim}N\left(t\right)\leq S_{0}.$$

It is straightforward to prove that for *ϵ* > 0 the simplex

(15) $$\Omega_{\epsilon }=\left\{\left(S,E,I,L\right)\in {\mathbb{R}}_{+}^{4};\;N\left(t\right)\leq \frac{\Lambda }{\mu }+\epsilon \right\}$$

is a compact invariant set for the system (2) and that for *ϵ* > 0 this set is absorbing. So, we limit our study to this simplex.

### 3.3. Basic Reproduction Ratio

Basic reproduction ratio is the average number of secondary cases produced by a single infective individual which is introduced into an entirely susceptible population.

We are going to compute the basic reproduction ratio of the system with control, and then deduce the basic reproduction ratio of the system without control.

**Proposition 4**

The basic reproduction ratio *R*_0_(*u*) of system (2), with control *u* = (*u*_1_, *u*_2_), is given by

(16) $$R_{0}\left(u\right)=\frac{\beta S_{0}}{R_{0,3}\left(u\right)}\left(R_{0,1}\left(u\right)+\delta R_{0,2}\left(u\right)\right),$$

where

(17) $$\begin{array}{cc} R_{0,1}\left(u\right) & =p_{2}k_{1}\left(1-r_{1}\right)\left(\gamma_{2}+\mu +d_{2}\right)+p_{1}\gamma_{2}\\ & \;\times \left[\mu +k_{1}\left(1-r_{1}\right)\right]+p_{2}\gamma_{1}\\ & \;\times \left(\mu +k_{1}+k_{2}-k_{2}u_{2}\left(t\right)\right)\left(1-r_{1}\right)\\ & \;+p_{1}\left(\gamma_{1}+\mu +d_{2}\right)\\ & \;\times \left[\mu +\left(k_{1}+k_{2}-k_{2}u_{2}\left(t\right)\right)\left(1-r_{1}\right)\right]\\ & \;+p_{3}\gamma_{2}k_{1}\left(1-r_{1}\right)+p_{3}\gamma_{1}\\ & \;\times \left[\mu +\left(k_{1}+k_{2}-k_{2}u_{2}\left(t\right)\right)\left(1-r_{1}\right)\right],\\ R_{0,2}\left(u\right) & =p_{3}\left[r_{2}\mu +\left(\mu +d_{1}\right)\left[\mu +k_{1}\left(1-r_{1}\right)\right]\right]\\ & \;+\Phi \left(1-u_{1}\left(t\right)\right)\left(1-r_{2}\right)\\ & \;\times \left(k_{1}+k_{2}-k_{2}u_{2}\left(t\right)\right)\left(1-r_{1}\right)\\ & \;+\mu \Phi \left(1-u_{1}\left(t\right)\right)\left(1-r_{2}\right)\left(p_{1}+p_{2}\right)\\ & \;+k_{2}\left(1-u_{2}\left(t\right)\right)\left(1-r_{1}\right)\\ & \;\times \left[r_{2}+\left(\mu +d_{1}\right)\left(p_{3}+p_{2}\right)\right],\\ R_{0,3}\left(u\right) & =\left(\mu +d_{2}\right)\left[\mu +d_{1}+\Phi \left(1-u_{1}\left(t\right)\right)\left(1-r_{2}\right)\right]\\ & \;\times \left[\mu +\left(k_{1}+k_{2}-k_{2}u_{2}\left(t\right)\right)\left(1-r_{1}\right)\right]\\ & \;+\gamma_{2}\mu \left[r_{2}+\mu +d_{1}+\Phi \left(1-u_{1}\left(t\right)\right)\left(1-r_{2}\right)\right]\\ & \;+r_{2}\left(\mu +d_{2}\right)\left[\mu +k_{2}\left(1-u_{2}\left(t\right)\right)\left(1-r_{1}\right)\right]\\ & \;+\gamma_{1}\left(\mu +d_{1}\right)\left[\mu +\left(k_{1}+k_{2}-k_{2}u_{2}\left(t\right)\right)\left(1-r_{1}\right)\right]\\ & \;+\gamma_{2}k_{1}\left(1-r_{1}\right)\left(\mu +d_{1}\right)+\gamma_{1}r_{2}\mu . \end{array}$$

**Proof**

The system (2) has an evident equilibrium (*S*_0_, 0,0, 0), where there is no disease. This equilibrium is the disease-free equilibrium (DFE). We calculate the basic reproduction ratio, *R*_0_(*u*), using the Van Den Driesseche and Watmough next generation approach [9] and the techniques reported in [10, 11]. In order to compute the basic reproduction ratio, it is important to distinguish new infections from all other class transitions in the population. The infected classes are *I*, *E*, and *L*. We can write system (2) as

(18) $$\dot{x}=ℱ\left(x\right)-𝒱\left(x\right),$$

where *x* = (*E*, *I*, *L*, *S*), *ℱ* is the rate of new infections in each class, *𝒱* ^+^ is the rate of transfer into each class by all other means, and *𝒱* ^−^(*x*) is the rate transfer out of each class. Hence,

(19) $$\begin{array}{c} \begin{array}{c} ℱ\left(x\right)={\left(\beta p_{2}\left(I+\delta L\right)S,\beta p_{1}\left(I+\delta L\right)S,\beta p_{3}\left(I+\delta L\right)S,0\right)}^{T},\\ 𝒱\left(x\right)=\left(\begin{array}{c} a_{11}E-r_{2}I-\gamma_{2}L\\ a_{22}I-k_{1}\left(1-r_{1}\right)E-\gamma_{1}L\\ a_{33}L-\phi \left(1-r_{2}\right)\left(1-u_{1}\right)I-a_{31}E\\ 0 \end{array}\right). \end{array} \end{array}$$

The Jacobian matrices of *ℱ* and *𝒱* at the disease-free equilibrium DFE can be partitioned as

(20) $$\begin{array}{c} Dℱ\left(\text{DFE}\right)=\left[\begin{array}{cc} F & 0\\ 0 & 0 \end{array}\right],\;\;\;D𝒱\left(\text{DFE}\right)=\left[\begin{array}{cc} V & 0\\ 0 & 0 \end{array}\right], \end{array}$$

where *F* and *V* correspond to the derivatives of *Dℱ* and *D𝒱* with respect to the infected classes:

(21) $$\begin{array}{c} F=\left(\begin{array}{ccc} 0 & \beta p_{2}S_{0} & \delta \beta p_{2}S_{0}\\ 0 & \beta p_{1}S_{0} & \delta \beta p_{1}S_{0}\\ 0 & \beta p_{3}S_{0} & \delta \beta p_{3}S_{0} \end{array}\right),\\ V=\left(\begin{array}{ccc} a_{11} & -r_{2} & -\gamma_{2}\\ -k_{1}\left(1-r_{1}\right) & a_{22} & -\gamma_{1}\\ -a_{31} & -a_{32} & a_{33} \end{array}\right). \end{array}$$

The basic reproduction ratio is defined, following Van den Driessche and Watmough [9], as the spectral radius of the next generation matrix, *FV* ^−1^.

From *R*_0_(*u*), we deduce *R*_0_(0) (basic reproduction ratio of the system without control) by taking *u* ≡ (0,0). We are going to compare *R*_0_(*u*) and *R*_0_(0).

**Note**

We have

(22) $$\begin{array}{c} R_{0,1}\left(u\right)=R_{0,1}\left(0\right)-\omega_{1}\left(u\right),\\ R_{0,2}\left(u\right)=R_{0,2}\left(0\right)-\omega_{2}\left(u\right),\\ R_{0,3}\left(u\right)=R_{0,3}\left(0\right)-\omega_{3}\left(u\right), \end{array}$$

where *ω*_1_, *ω*_2_, and *ω*_3_ are nonnegative functions defined by

(23) $$\begin{array}{cc} \omega_{1}\left(u\right) & =\left(1-r_{1}\right)\left[\gamma_{1}+p_{1}\left(\mu +d_{2}\right)\right]k_{2}u_{2},\\ \omega_{2}\left(u\right) & =\phi \left(1-r_{2}\right)\left[k_{2}u_{2}+u_{1}\left(k_{1}+k_{2}-k_{2}u_{2}\right)\right]\\ & \;\times \left(1-r_{1}\right)+\phi \mu \left(1-r_{2}\right)\left(p_{1}+p_{2}\right)u_{1}\\ & \;+k_{2}\left(1-r_{1}\right)\left[r_{2}+\left(\mu +d_{1}\right)\left(p_{3}+p_{2}\right)\right]u_{2},\\ \omega_{3}\left(u\right) & =\left(\mu +d_{2}\right)\phi \left(1-r_{2}\right)\\ & \;\times \left[k_{2}u_{2}\left(1-r_{1}\right)+u_{1}\left[\mu +\left(k_{1}+k_{2}-k_{2}u_{2}\right)\left(1-r_{1}\right)\right]\right]\\ & \;+k_{2}\left(1-r_{1}\right)u_{2}\left[\gamma_{1}\left(\mu +d_{1}\right)+r_{2}\left(\mu +d_{2}\right)\right]\\ & \;+\gamma_{2}\mu \phi \left(1-r_{2}\right)u_{1}. \end{array}$$

**Remark 5**

Note that

(24) $$\begin{array}{c} R_{0}\left(u\right)-R_{0}\left(0\right)=\frac{\omega_{3}\left(u\right)}{R_{0,3}\left(u\right)}\left[R_{0}\left(0\right)-\frac{\omega_{1}\left(u\right)+\delta \omega_{2}\left(u\right)}{\omega_{3}\left(u\right)}\right]. \end{array}$$

We can remark that in some conditions, depending only on system parameters, we can have

(25) $$\begin{array}{c} R_{0}\left(u\right)\leq R_{0}\left(0\right). \end{array}$$

**Remark 6**

Let us examine sensitivity of the basic reproduction ratio without control *R*_0_(0) with respect to *β*. It is easy to prove that

(26) $$\begin{array}{c} \frac{\partial R_{0}\left(0\right)}{\partial \beta }=\frac{S_{0}}{R_{0,3}\left(0\right)}\left(R_{0,1}\left(0\right)+\delta R_{0,2}\left(0\right)\right)>0. \end{array}$$

Thus, *R*_0_(0) increases with *β*.

### 3.4. Equilibria

The equilibrium (*S*, *Y*) on system (2) can be obtained by setting the right-hand side of all the equations in model (4) equal to zero, that is,

(27) $$\begin{array}{c} \varphi \left(S\right)-S\langle \eta ,Y\rangle =0,\\ S\langle \eta ,Y\rangle B+A\left(t\right)Y=0. \end{array}$$

From the second equation of (27), one has *Y* = *S*(−*A* ^−1^(*t*))〈*η*, *Y*〉*B*. And replacing in 〈*η*, *Y*〉 yields

(28) $$\begin{array}{c} \langle \eta ,Y\rangle =S\langle \eta ,\left(-A^{-1}\left(t\right)\right)B\rangle \langle \eta ,Y\rangle . \end{array}$$

The case 〈*η*, *Y*〉 = 0 implies *φ*(*S*) = 0 and *A*(*t*)*Y* = 0. Since *A* is nonsingular, this gives the disease-free equilibrium *P* ^0^ = (*S*_0_, 0,0, 0).

The case 〈*η*, *Y*〉 ≠ 0 implies S* = *S*_0_/*R*_0_(*u*). From (28), we have Y* = (E*,I*,L*)^*T*^ = (−*A* ^−1^(*t*))*Bφ*(S*).

After calculations, we obtained that, with *R*_0_(*u*) > 1, the model (4) has a unique endemic equilibrium P*(*u*) = (S*(*u*), E*(*u*), I*(*u*), L*(*u*)) given by

(29) $$\begin{array}{c} \begin{array}{c} S^{*}\left(u\right)=\frac{S_{0}}{R_{0}\left(u\right)},\\ E^{*}\left(u\right)=\frac{Q_{1}\left(u\right)\Lambda }{R_{0}^{3}\left(u\right)}\left(1-\frac{1}{R_{0}\left(u\right)}\right),\\ I^{*}\left(u\right)=\frac{Q_{2}\left(u\right)\Lambda }{R_{0}^{3}\left(u\right)}\left(1-\frac{1}{R_{0}\left(u\right)}\right),\\ L^{*}\left(u\right)=\frac{Q_{3}\left(u\right)\Lambda }{R_{0}^{3}\left(u\right)}\left(1-\frac{1}{R_{0}\left(u\right)}\right), \end{array} \end{array}$$

where

(30) $$\begin{array}{cc} Q_{1}\left(u\right) & =p_{1}r_{2}\left(\gamma_{1}+\gamma_{2}+\mu +d_{2}\right)+\left(r_{2}+\mu +d_{1}\right)\left(p_{2}\gamma_{1}+p_{3}\gamma_{3}\right)\\ & \;+p_{3}r_{2}\gamma_{1}+\gamma_{2}\phi \left(1-u_{1}\left(t\right)\right)\left(1-r_{2}\right)\left(p_{1}+p_{3}\right)\\ & \;+p_{2}\left(\gamma_{2}+\mu +d_{2}\right)\left[r_{2}+\mu +d_{1}+\phi \left(1-r_{2}\right)\right],\\ Q_{2}\left(u\right) & =p_{2}k_{1}\left(1-r_{1}\right)\left(\gamma_{2}+\mu +d_{2}\right)+p_{1}\gamma_{2}\left[\mu +k_{1}\left(1-r_{1}\right)\right]\\ & \;+p_{2}\gamma_{1}\left(k_{1}+k_{2}-k_{2}u_{2}\left(t\right)\right)\left(1-r_{1}\right)+p_{3}\gamma_{2}k_{1}\left(1-r_{1}\right)\\ & \;+p_{1}\left(\gamma_{1}+\mu +d_{2}\right)\left[\mu +\left(k_{1}+k_{2}-k_{2}u_{2}\right)\left(1-r_{1}\right)\right]\\ & \;+p_{3}\gamma_{1}\left[\mu +\left(k_{1}+k_{2}-k_{2}u_{2}\left(t\right)\right)\left(1-r_{1}\right)\right],\\ Q_{3}\left(u\right) & =p_{3}\left[r_{2}\mu +\left(\mu +d_{1}\right)\left(\mu +k_{1}\left(1-r_{1}\right)\right)\right]+\phi \left(1-u_{1}\left(t\right)\right)\\ & \;\times \left[\left(k_{1}+k_{2}-k_{2}u_{2}\left(t\right)\right)\left(1-r_{1}\right)\right]\\ & \;+\phi \left(1-u_{1}\left(t\right)\right)\mu \left(p_{1}+p_{3}\right)+k_{2}\left(1-u_{2}\left(t\right)\right)\\ & \;\times \left(1-r_{1}\right)\left[r_{2}+\left(\mu +d_{1}\right)\left(p_{2}+p_{3}\right)\right]. \end{array}$$

**Lemma 7**

When *R*_0_(*u*) > 1, model (2) has a unique endemic equilibrium defined as in (29).

**Remark 8**

It is showed in [7] that

- if *R*_0_(0) ≤ 1, the disease-free equilibrium *P* ^0^ is globally asymptotically stable on the nonnegative orthant ℝ_4_^+^. This means that, the disease naturally dies out in the host population;
- If *R*_0_(0) > 1, then the positive endemic equilibrium state P*(0) of model (2) is globally asymptotically stable on the set *Ω*_*ϵ*_ when

(31) $$\begin{array}{c} \left(\alpha_{4}+\delta \alpha_{2}\right)\gamma_{1}Q_{3}\left(0\right)=\left(\alpha_{3}+\delta \alpha_{1}\right)Q_{2}\left(0\right)p_{3}\beta S^{*}\left(0\right),\\ \begin{array}{cc} & \left(\mu +\left(k_{1}+k_{2}\right)\left(1-r_{1}\right)\right)k_{2}\left(1-r_{1}\right)Q_{1}\left(0\right)\\ & \;=\left[k_{1}\left(1-r_{1}\right)\frac{\alpha_{4}+\delta \alpha_{2}}{\alpha_{3}+\delta \alpha_{1}}+k_{2}\left(1-r_{1}\right)\right]Q_{3}\left(0\right)p_{2}\beta \delta S^{*}\left(0\right), \end{array} \end{array}$$

with

(32) $$\begin{array}{cc} \alpha_{1} & =\left[\mu +\left(k_{1}+k_{2}\right)\left(1-r_{1}\right)\right]\left[\mu +d_{1}+\phi \left(1-r_{2}\right)\right]\\ & \;+r_{2}\left[\mu +k_{2}\left(1-r_{1}\right)\right],\\ \alpha_{2} & =\left[\mu +\left(k_{1}+k_{2}\right)\left(1-r_{1}\right)\right]\phi \left(1-r_{2}\right)+k_{2}\left(1-r_{1}\right)r_{2},\\ \alpha_{3} & =\left[\mu +\left(k_{1}+k_{2}\right)\left(1-r_{1}\right)\right]\gamma_{1}+\gamma_{2}k_{1}\left(1-r_{1}\right),\\ \alpha_{4} & =\left[\mu +\left(k_{1}+k_{2}\right)\left(1-r_{1}\right)\right]\left(\gamma_{1}+\mu +d_{2}\right)\\ & \;+\gamma_{2}\left[\mu +k_{1}\left(1-r_{1}\right)\right]. \end{array}$$

## 4. Optimal Control

### 4.1. Definition of the Cost Function

Let *B*_*i*_, *i* = 1,2, be the cost associated to the control *u*_*i*_(*t*), *i* = 1,2. (*B*_*i*_ represents the necessary means to realize the control defined by *u*_*i*_). Our cost function is hence

(33) $$\begin{array}{c} J\left(u_{1},u_{2}\right)=\overset{t_{f}}{\underset{0}{\int }}\left[L\left(t\right)+\overset{2}{\underset{i=1}{\sum }}\frac{B_{i}}{2}u_{i}^{2}\left(t\right)\right]dt. \end{array}$$

The cost function is defined having in mind that, we are going to penalize the number of lost sight person. This justifies the presence of the term *L*.

The problem now is to find u* = (*u*_1_*, *u*_2_*) satisfying

(34) $$\begin{array}{c} J\left(u_{1}^{*},u_{2}^{*}\right)=\underset{\Omega }{\min}\;J\left(u_{1},u_{2}\right), \end{array}$$

where *Ω* = {(*u*_1_, *u*_2_) ∈ *L* ^1^(*o*, *t*_*f*_); *a*_*i*_ ≤ *u*_*i*_ ≤ *b*_*i*_, *i* = 1,2} and *a*_*i*_, *b*_*i*_ are nonnegative constants such that *a*_*i*_, *b*_*i*_ ∈ [0,1].

### 4.2. Resolution of the Optimal Problem

Using the Pontryagin's maximum principle [12], problems (2)–(34) are reduced to minimize the function *H* defined by

(35) $$\begin{array}{c} H\left(u,S,E,I,L\right)=L\left(t\right)+\frac{1}{2}\overset{2}{\underset{j=1}{\sum }}B_{j}u_{j}^{2}\left(t\right)+\overset{4}{\underset{i=1}{\sum }}\lambda_{i}g_{i}, \end{array}$$

where the functions (*g*_*i*_, *i* = 1,2, 3,4) are defined by (2).

The necessary conditions for the existence of the solution for problem (34) are

(36) $$\begin{array}{c} \frac{\partial \lambda_{1}}{\partial t}=-\frac{\partial H}{\partial S},\\ \frac{\partial \lambda_{2}}{\partial t}=-\frac{\partial H}{\partial E},\\ \frac{\partial \lambda_{3}}{\partial t}=-\frac{\partial H}{\partial I},\\ \frac{\partial \lambda_{4}}{\partial t}=-\frac{\partial H}{\partial L}, \end{array}$$

(37) $$\begin{array}{c} \frac{\partial H}{\partial u_{i}}=0\;\left(i=1,2\right). \end{array}$$

System (36) leads to the adjoint system:

(38) $$\begin{array}{c} \dot{\lambda }\left(t\right)={\left(0,0,0,-1\right)}^{T}+\Gamma \left(t\right)\lambda \left(t\right), \end{array}$$

with *λ*(*t*) = (*λ*_*i*_(*t*))_*i*∈{1,2,3,4}_, and Γ(*t*) = (Γ_*ij*_)_1≤*i*,*j*≤4_ is a nonconstant 4 × 4 matrix defined as

(39) $$\begin{array}{cc} & \Gamma_{11}=\mu S+\beta \left(I+\delta L\right),\\ & \Gamma_{12}=-\beta p_{2}\left(I+\delta L\right),\\ & \Gamma_{13}=-\beta p_{1}\left(I+\delta L\right),\\ & \Gamma_{14}=-\beta p_{3}\left(I+\delta L\right),\\ & \Gamma_{21}=0,\\ & \Gamma_{22}=\mu +\left(k_{1}+k_{2}-k_{2}u_{2}\left(t\right)\right)\left(1-r_{1}\right),\\ & \Gamma_{23}=-k_{1}\left(1-r_{1}\right),\\ & \Gamma_{24}=-k_{2}\left(1-u_{2}\right)\left(1-r_{1}\right),\\ & \Gamma_{31}=\beta S,\\ & \Gamma_{32}=-\left(\beta p_{2}S+r_{2}\right),\\ & \Gamma_{33}=r_{2}+\mu +d_{1}+\Phi \left(1-u_{1}\left(t\right)\right)\left(1-r_{2}\right)-\beta p_{1}S,\\ & \Gamma_{34}=-\Phi \left(1-u_{1}\left(t\right)\right)\left(1-r_{2}\right)-\beta p_{3}S,\\ & \Gamma_{41}=\beta \delta S,\\ & \Gamma_{42}=-\left(\beta p_{2}\delta S+\gamma_{2}\right),\\ & \Gamma_{43}=-\left(\gamma_{1}+\beta p_{1}\delta S\right),\\ & \Gamma_{44}=\gamma_{1}+\gamma_{2}+\mu +d_{2}-\beta p_{3}\delta S, \end{array}$$

with transversality conditions

(40) $$\begin{array}{c} \lambda_{i}\left(t_{f}\right)=0;\;i\in \left\{1,2,3,4\right\}. \end{array}$$

**Remark 9**

The transversality conditions are due to the fact that after the period of control (*t*_*f*_), there is no more information given by the adjoint system.

**Proposition 10**

System (37) leads to

(41) $$\begin{array}{c} \begin{array}{c} u_{1}^{*}\left(t\right)=\min\left\{\max\left(a_{1};\frac{\phi \left(1-r_{2}\right)I}{B_{1}}\left(\lambda_{4}-\lambda_{3}\right)\right);b_{1}\right\},\\ u_{2}^{*}\left(t\right)=\min\left\{\max\left(a_{2};\frac{k_{2}\left(1-r_{1}\right)E}{B_{2}}\left(\lambda_{4}-\lambda_{2}\right)\right);b_{2}\right\}. \end{array} \end{array}$$

**Proof**

The existence of an optimal control pair is due to the convexity of integrand of *J* with respect to (*u*_1_, *u*_2_), a *priori* boundedness of the state solutions, and the *Lipschitz* property of the state system with respect to the state variables [12]. By considering the optimality conditions (37), and solving for *u*_1_*, *u*_2_*, subject to the constraints, the characterizations (41) are derived. To illustrate the characterization of *u*_1_*, we have

(42) $$\begin{array}{c} \frac{\partial H}{\partial u_{1}}=B_{1}u_{1}+\phi \left(1-r_{2}\right)I\left(\lambda_{3}-\lambda_{4}\right)=0, \end{array}$$

at *u*_1_* on the set {*t*/*a*_1_ < *u*_1_*(*t*) < *b*_1_}. On this set,

(43) $$\begin{array}{c} u_{1}^{*}\left(t\right)=\frac{\phi \left(1-r_{2}\right)}{B_{1}}I\left(\lambda_{4}-\lambda_{3}\right). \end{array}$$

Taking into account the bounds on *u*_1_*, we obtain the characterization of *u*_1_* in (41).

### 4.3. Determination of the Control Function

In this section, we are going to show step by step, how to determine the optimal functions numerically.

**Remark 11**

The main difficulty here for the optimal control is that we have initial conditions for system (2) and final conditions for the adjoint system (transversality conditions).

To overcome this difficulty, we proceed as follows.

**Step 1**

We choose a control function *u*(*t*) ≡ *u* ^*c*^(*t*) in the set *Ω*. However, this choice is not a random process; it depends on the strategy we need to adopt. For example, in this paper, we adopt a strategy which is very strict at the beginning of the control. We choose

(44) $$\begin{array}{c} \begin{array}{cccc} u_{1}^{c}\left(t\right)=b_{1}, & & u_{2}^{c}\left(t\right)=b_{2}, & \;\forall t\in \left[0,t_{f}\right] \end{array}. \end{array}$$

**Step 2**

Then, with this choice of the control function *u* ^*c*^(*t*), one determines the solution (*S*(*t*), *E*(*t*), *I*(*t*), *L*(*t*)) of the Cauchy problem associated to system (2).

**Step 3**

The knowledge of *u*(*t*) ≡ *u* ^*c*^(*t*) and (*S*(*t*), *E*(*t*), *I*(*t*), *L*(*t*)) allows us to determine the solution *λ*(*t*) of the Cauchy problem associated to the adjoint system with transversality conditions. This takes us to the control functions defined in (41) by u* = (*u*_1_*, *u*_2_*).

**Step 4**

For one thing we have the chosen control function *u* ^*c*^, for another thing we have the control function u*. We take a convex combination of those functions as follows:

(45) $$\begin{array}{c} u\left(t\right)=\left(1-\frac{t}{t_{f}}\right)u^{c}\left(t\right)+\frac{t}{t_{f}}u^{*}\left(t\right) \end{array}$$

for *t* ∈ [0, *t*_*f*_].

**Step 5**

This process is repeated (Steps 2, 3, and 4), and iterations are stopped when the values at the unknown iteration are very closed to the ones at the present iteration.

## 5. Numerical Simulations

We are going to provide numerical simulations to illustrate our studies.

**Figure 3:**
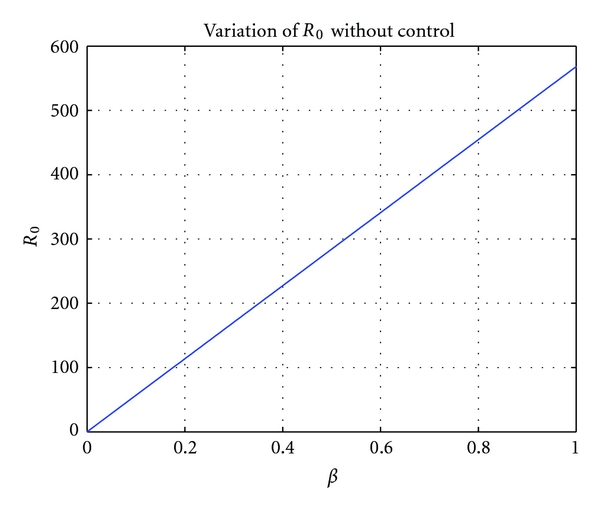
Variation of the basic reproduction ratio without control as a function of *β*, with *ϕ* = 0.0022 and *k*_2_ = 0.0006.

We assumed that *β* is variable because it strongly influences the basic reproduction ratio (Remark 6). This is illustrated by Figure 3. 

We also assume that the parameters *ϕ* and *k*_2_, which denote the rate of progression from infectious to lost to follow up and the rate of progression from latently infected to lost follow up, respectively, are variable just to highlight the fact that the optimal control depends on that parameters.

For numerical simulations the values of the above parameters are *β* ∈ {0.002; 0.003; 0.02}, *ϕ* ∈ {0.0022; 0.1; 0.5}, and *k*_2_ ∈ {0.0006; 0.006}. The values of the other parameters are given in Table 1.

We solve the state equation (2) with the chosen functions *u*_*i*_ = *u*_*i*_^*c*^ (*i* = 1,2) using the Runge-Kutta forward scheme of order 4. Then, we solve the adjoint system using the backward Runge-Kutta scheme of order 4.

We deduce *u*_i_* (*i* = 1,2) from system (41).

For those simulations, we take *t*_*f*_ = 5 years as control period. We also assume that the total population number is *N* = 500 individuals subdivided as follows: *S*(0) = 50, *E*(0) = 100, *I*(0) = 150, and *L*(0) = 200.

**Figure 4:**
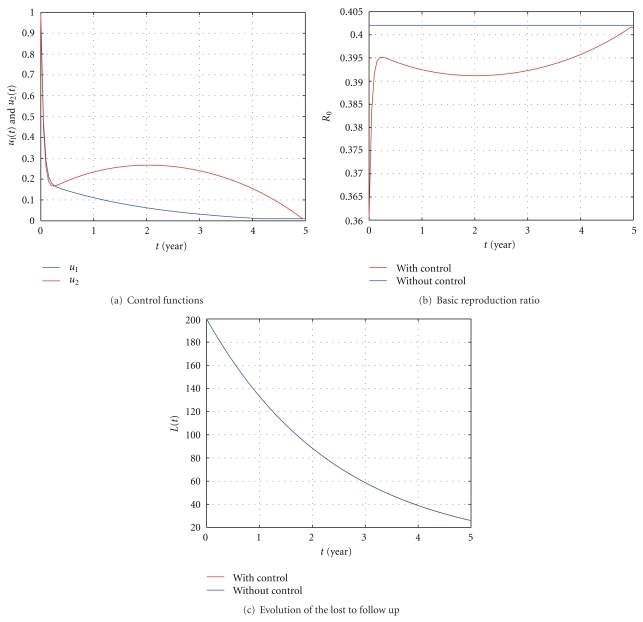
The influence of the control with *S*(0) = 50, *E*(0) = 100, *I*(0) = 150, *L*(0) = 200, *β* = 0.002, *ϕ* = 0.0022, and *k*_2_ = 0.0006. All the other parameter values are as in Table 1.

In Figure 4, *β* = 0.002 is chosen to assure that the reproduction ratio *R*_0_ without control is less than 1. The values of *ϕ* and *k*_2_ are chosen here small enough to show that the control would not really be necessary (Figure 4(a)).  Figure 4(b): the average basic reproduction ratio is about 0.4020 without control and about 0.3974 with it. This is due to the fact that our control is not rigorous enough.  Figure 4(c): the average number during *t*_*f*_ = 5 years of lost to follow up is about 86.4411 individuals without control. This value is approximately the same with control 86.3869. This is because the rate at which infectious becomes lost to follow up *ϕ* = 0.0022 and the rate at which latently infected becomes lost to follow up *K*_2_ = 0.0006 are very small.

**Figure 5:**
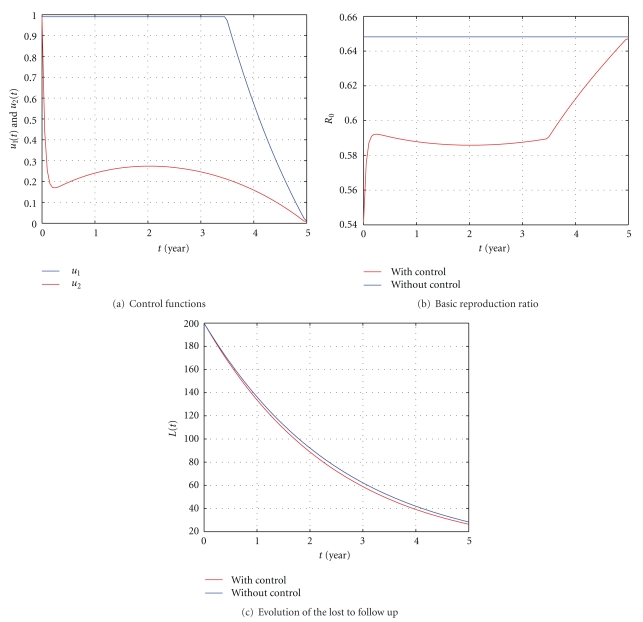
The influence of the control with *S*(0) = 50, *E*(0) = 100, *I*(0) = 150, *L*(0) = 200, *β* = 0.003, *ϕ* = 0.1, and *k*_2_ = 0.0006. All the other parameter values are as in Table 1.

In Figure 5, *β* = 0.003 is chosen to assure that the reproduction ratio *R*_0_ without control is less than 1. The value of *k*_2_ is chosen here small enough to show that the associated control function *u*_2_ would not really be necessary. Unlike the value of *ϕ* which the associated control function *u*_1_ is strict (Figure 5(a)).  Figure 5(b): the average basic reproduction ratio is about 0.6482 without control and about 0.6033 with it.  Figure 5(c): the average number during *t*_*f*_ = 5 years of lost to follow up is about 89.4644 individuals without control and about 86.7582 with it. In a period of *t*_*f*_ = 5 years of control, we succeed in keeping about 3 infectious individuals in a care center.

**Figure 6:**
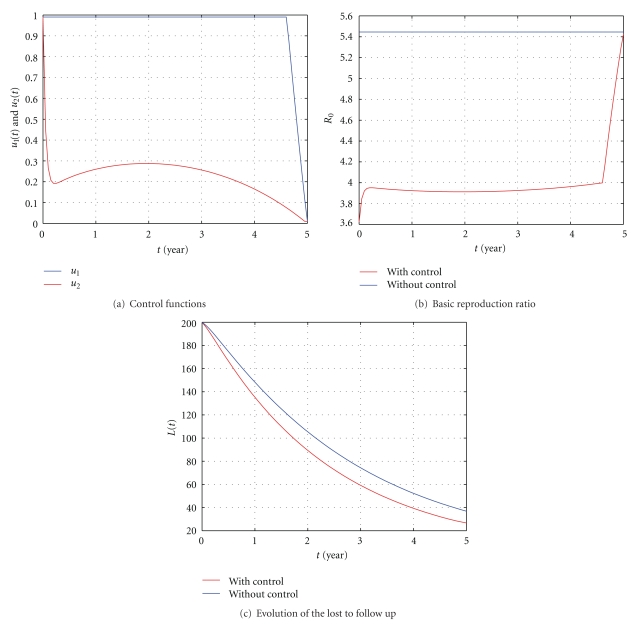
The influence of the control with *S*(0) = 50, *E*(0) = 100, *I*(0) = 150, *L*(0) = 200, *β* = 0.02, *ϕ* = 0.5, and *k*_2_ = 0.0006. All the other parameter values are as in Table 1.

In Figure 6, *β* = 0.02 is chosen to assure that the reproduction ratio *R*_0_ without control is greater than 1. The value of *k*_2_ is chosen here small enough to show that the associated control function *u*_2_ would not really be necessary. Unlike the value of *ϕ* which the associated control function *u*_1_ is very strict during the whole control period (Figure 6(a)).  Figure 6(b): the average basic reproduction ratio is about 5.4460 without control and about 4.0424 with it.  Figure 6(c): the average number during *t*_*f*_ = 5 years of lost to follow up is about 100.4334 individuals without control and about 87.5361 with it. In a period of *t*_*f*_ = 5 years of control, we succeed in keeping about 13 infectious individuals in a care center.

**Figure 7:**
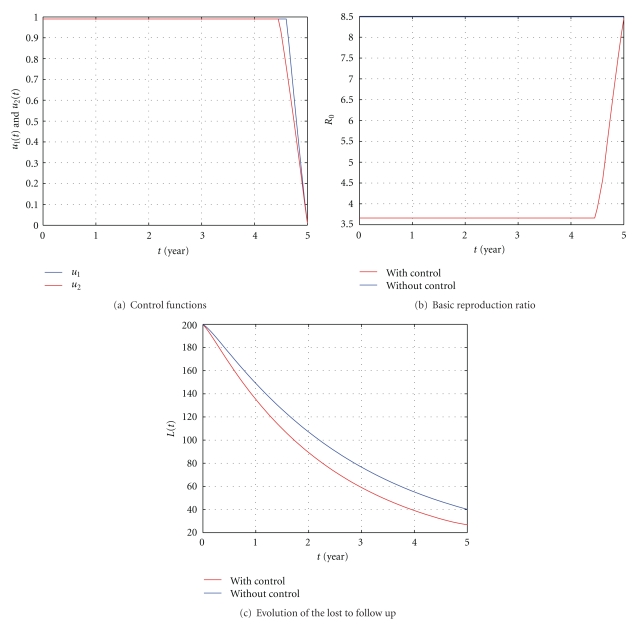
The influence of the control with *S*(0) = 50, *E*(0) = 100, *I*(0) = 150, *L*(0) = 200, *β* = 0.02, *ϕ* = 0.5, and *k*_2_ = 0.006. All the other parameter values are as in Table 1.

In Figure 7, *β* = 0.02 is chosen to assure that the reproduction ratio *R*_0_ without control is greater than 1. The values of *k*_2_ and *ϕ* are chosen in order to make both control functions *u*_1_ and *u*_2_ strict (Figure 7(a)).  Figure 7(b): the average basic reproduction ratio is about 8.4875 without control and about 3.9675 with it. The basic reproduction ratio without control is about twice as large as the one with control.  Figure 7(c): the average number during *t*_*f*_ = 5 years of lost to follow up is about 102.3067 individuals without control and about 87.4159 with it. In a period of *t*_*f*_ = 5 years of control, we succeed in keeping about 15 infectious individuals in a care center.

## 6. Summary and Discussion

This has considered the problem of optimal control of the transmission dynamics of TB. A model considering a new class has been investigated and analyzed. An optimal control strategy has been presented, and the results show how important it is to control the lost to follow up class, which is very crucial to the study of the disease. Numerical simulations have been given to illustrate the effectiveness and efficiency of the proposed control scheme. In Africa, it is very important to keep infectious individuals in a care center in order to complete their treatment and avoid the quick transmission of the disease. Our control strategy helps to do so, though other control strategies could be investigated.

For discussion, it should be noted that the model investigated here is based on some restrictive assumptions as an epidemic model. We have assumed that

1. any recruitment, is into the susceptible class and occur at a constant rate Λ,
2. we have not taken into account the class of recovered individuals.

The first assumption is met for the dynamical study of a host population evolving in a restrictive domain. To overpass this assumption, we could introduce the diffusion phenomenon in the model.

The second is due to the fact that the complete recovering from TB is just apparent in general [13]. In other words, some infectious individuals apparently recover but actually harbor TB bacteria, which are in an inactive state. Thus, those TB bacteria are undetectable by the antibodies or other molecules aiming to fight the disease.

## Figures and Tables

**Table 1:** Table of parameter values [14–16].

Parameters	Description	Estimated values	Source
Λ	Recruitment rate of susceptible individuals	5 (yr)^−1^	Assumed
*β*	Transmission rate	variable	Assumed
*μ*	Natural death rate	0.019896 (yr)^−1^	[14]
*d*_1_	TB-induced mortality for the follow up	0.02272 (yr)^−1^	[15]
*d*_2_	TB-induced mortality for the lost to follow up	0.20 (yr)^−1^	[15]
*δ*	Fraction of lost to follow up that are still infectious	1 (yr)^−1^	Assumed
*ϕ*	Rate at which infectious become lost to follow up	Variable	Assumed
*p*_1_	Fast route to infectious class	0.3 (yr)^−1^	[15]
*p*_3_	Fast route to lost to follow up class	0.1 (yr)^−1^	Assumed
*r*_1_	Chemoprophylaxis of latently infected individuals	0.001 (yr)^−1^	[15]
*r*_2_	Recovery rate of the infectious	0.7311 (yr)^−1^	[15]
*γ*_1_	Rate at which the lost to follow up return to the hospital	0.2 (yr)^−1^	Assumed
*γ*_2_	Recovering rate for the lost to follow up	0.001 (yr)^−1^	Assumed
*k*_1_	Rate of progression from infected latently to infectious	0.0005 (yr)^−1^	[16]
*k*_2_	Rate of progression from infected latently to lost to follow up	Variable	Assumed
